# High yield exogenous protein HPL production in the *Bombyx mori* silk gland provides novel insight into recombinant expression systems

**DOI:** 10.1038/srep13839

**Published:** 2015-09-15

**Authors:** Huan Wang, Lu Wang, Yulong Wang, Hui Tao, Weimin Yin, Yanghu SiMa, Yujun Wang, Shiqing Xu

**Affiliations:** 1 School of Biology and Basic Medical Sciences, Medical College, Soochow University, Suzhou 215123, China; 2 R&D Division, Okamoto Corporation, Nara 635-8550, Japan; 3 National Engineering Laboratory for Modern Silk, Soochow University, Suzhou 215123, China

## Abstract

The silk gland of *Bombyx mori* (BmSG) has gained significant attention by dint of superior synthesis and secretion of proteins. However, the application of BmSG bioreactor is still a controversial issue because of low yields of recombinant proteins. Here, a 3057 bp full-length coding sequence of *Hpl* was designed and transformed into the silkworm genome, and then the mutant (*Hpl*/*Hpl*) with specific expression of *Hpl* in posterior BmSG (BmPSG) was obtained. In the mutants, the transcription level of *Fib-L* and *P25*, and corresponding encoding proteins, did not decrease. However, the mRNA level of *Fib-H* was reduced by 71.1%, and Fib-H protein in the secreted fibroin was decreased from 91.86% to 71.01%. The mRNA level of *Hpl* was 0.73% and 0.74% of *Fib-H* and *Fib-L*, respectively, while HPL protein accounted for 18.85% of fibroin and 15.46% of the total amount of secreted silk protein. The exogenous protein was therefore very efficiently translated and secreted. Further analysis of differentially expressed gene (DEG) was carried out in the BmPSG cells and 891 DEGs were detected, of which 208 genes were related to protein metabolism. Reduced expression of endogenous silk proteins in the BmPSG could effectively improve the production efficiency of recombinant exogenous proteins.

*Bombyx mori* is the main target insect of human domestication and feeding selection in China, India, Uzbekistan, Thailand, and Brazil, and the sericulture industry utilizes silkworms for the efficient production of silk proteins as an important economic source for more than 30 million households. The silk gland of *Bombyx mori* (BmSG) is a highly specialized organ that has an excellent ability to synthesize and secrete proteins. At the larval stage, the weight of the BmSG increases rapidly by 15,0000 times in 3.5 weeks, while almost pure proteins are synthesized and secreted, accounting for up to 25% of the body weight. A BmSG synthesizes 6 × 10^9^ silk fibroin proteins per second, which is more than 60 times faster than the rate of a human liver cell synthesizing serum albumin^1^,^2^.

Although BmSG was not recognized as a major recombinant protein production platform^3^,^4^,^5^, it could be the perfect host system for exogenous protein production because transgenic silkworms can be efficiently produced using piggyBac vectors^6^,^7^, recombinant protein production can be targeted to the BmSG with tissue specific promoters^8^,^9^,^10^,^11^,^12^,^13^, and the BmSG is naturally equipped to assemble exogenous proteins into secreted silk proteins^14^. Previous reports demonstrated the production of collagen^9^,^15^,^16^, human pharmaceutical proteins^17^,^18^,^19^,^20^,^21^, and other proteins^11^,^22^,^23^ in BmSG.

Many studies using transgenic silk glands as recombinant protein production systems have encountered problems of low efficiency expression and secretion of exogenous proteins (see Supplementary Table S1 online)^24^,^25^,^26^,^27^,^28^,^29^,^30^,^31^,^32^,^33^,^34^,^35^,^36^,^37^,^38^,^39^. Even though the most powerful promoter of the fibroin heavy chain was used, the ratio of recombinant protein to endogenous proteins never exceeded 15%^37^,^40^. Although introduction of three or more foreign genes into the silkworm genome^21^, combinations of enhancers (hr3/IE1)^16^, and the improvement of strategies for vector construction^33^ have significantly increased the expression of fusion proteins, the expression levels of exogenous proteins was less than the expression of silk proteins. A popular explanation is that the BmSG has been already highly adapted to silk protein synthesis during the specialization process, but the ability of synthesizing other endogenous and exogenous proteins has significantly degenerated.

In our laboratory previous work, we found an interesting phenomenon through observing the degenerated BmSG cells during the process of silkworm pupation, when silk proteins synthesis had stopped, the BmPSG began to efficiently synthesize the reproductive storage protein 30 K^41^, suggesting that high-efficiency protein synthesis functions of the BmSG cells could be used for the synthesis of exogenous proteins, if the silk protein genes were knocked down or knocked out. Based on this objective, Wang and Nakagaki^42^ successfully constructed a *Fib-H* deficiency system by knocking out the heavy chain gene (*Fib-H*) of silk fibroin. Fib-H protein synthesis in the BmPSG cells indeed stopped, but the secretion of the other two components of fibroin proteins, light chain (Fib-L), and fibrohexamerin (BmFhx/P25) was completely inhibited in the BmPSG. Then the expression of exogenous macromolecular spider silk proteins was achieved in the above *Fib-H* knockout system, and no secretion of exogenous proteins in the glandular body and outside of the body were observed (unpublished data). Therefore, elucidation of the regulation mechanisms, from conversion of efficient silk protein synthesis to the synthesis of other proteins in the BmSG cells, has become the focus of current studies. This study focused on two questions: 1) Can a silkworm BmPSG system be established in which Fib-H expression is down-regulated, while at the same time maintaining the synthesis and secretion of Fib-L and P25 and achieving efficient secretion of exogenous proteins? and 2) What are the characteristic changes in genome expression of the BmSG tissues which allow more efficient secretion of exogenous proteins?

## Results

### Transgenic silkworm system TBH (*Hpl*/*Hpl*)

**Figure 1:**
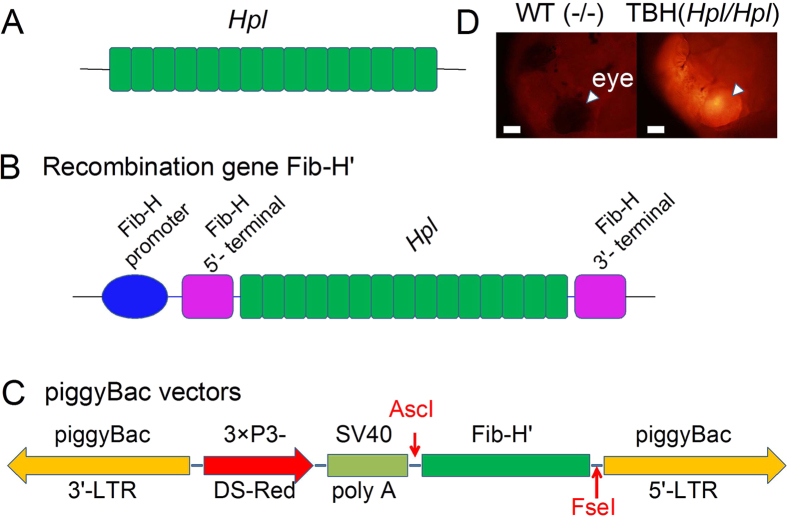
Vector construction. (**A**) The artificial sequence of *Hpl*, with full-length 3057 bp, is shown. (**B**) The recombinant gene *Fib-H*′. *Fib-H*′ was controlled by the *Fib-H* promoter. The *Fib-H* signal peptide sequence was added to the 5′ end, and the 3′ terminal sequence of silkworm *Fib-H* gene was added to the 3′ end. (**C**) The gene transfer vector. (**D**) The RFP-positive pupa. Bars are 1mm.

A gene transfer vector for expression of exogenous protein HPL in the BmPSG was constructed according to the steps in Fig. 1, and based on the method of Wen *et al.*^43^. To enhance the secretion of expressed protein HPL from the silk gland cells, the promoter and the 5′ terminus of the signal peptide sequence, and the subsequent 403 base pair (bp) sequence of the silkworm *Fib-H* gene were linked upstream of *Hpl*, while the 3′ terminal sequence of the silkworm *Fib-H* gene was linked downstream, thus the *Hpl* gene was reconstructed and named as *Fib-H*′ (Fig. 1A,B). An artificial promoter 3 × P3 that consisted of three tandem silkworm eye and nervous system specific transcription factor PAX-6 binding sequences was used to control the ERFP (Ds-Red) reporter gene, and the gene transfer vector was constructed (Fig. 1C). RFP-positive individuals of the G0 generation after injection at the egg stage were passaged by self-fertilization. Individual silkworms with red fluorescent eyes at the 3rd–5th larval instar and pupal stage were identified as a transgenic G1 generation. The *Hpl* sequence was verified in adults after spawning, and individuals were continuously screened to the G6 generation by the characteristics of red fluorescent eyes (Fig. 1D). Therefore, a genetically stable *Hpl* transfer system TBH (*Hpl*/*Hpl*) was obtained.

### Expression analysis of exogenous protein HPL

**Figure 2:**
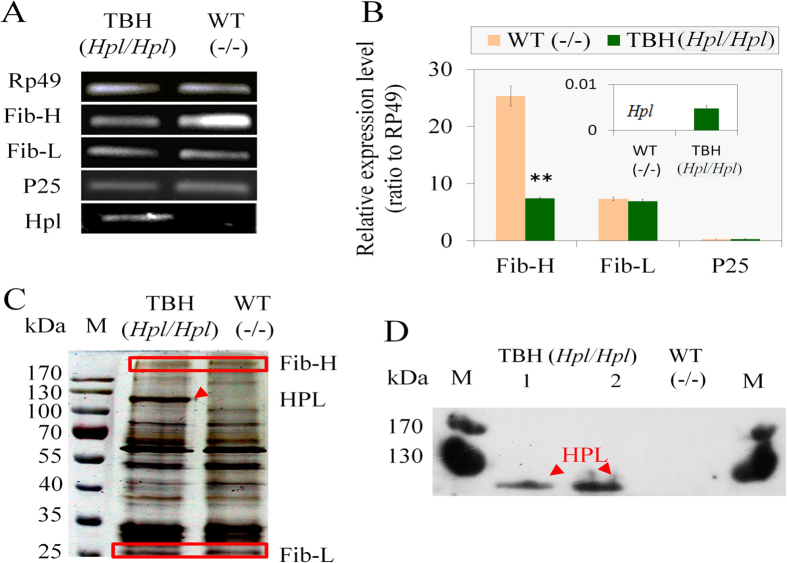
RT-PCR (A), Real-time PCR (B), SDS-PAGE (C), and western blotting (D). RNA was isolated from the BmPSG at the wandering stage, and used for RT-PCR and Real-time PCR. Proteins were extracted from the cocoon shells using the method of Teulé *et al.*^31^ and used for SDS-PAGE and western blotting. The red arrows in figures C and D show the foreign protein HPL (MS is approx 120 kDa).

The results of reverse transcriptase-polymerase chain reaction (RT-PCR) (Fig. 2A) and real-time PCR (Fig. 2B) demonstrated that the exogenous gene *Hpl* (*Fib-H*′) was successfully expressed in the BmPSG of TBH. The results of sodium dodecyl sulfate–polyacrylamide gel electrophoresis (SDS-PAGE) (Fig. 2C) and western blotting (Fig. 2D) further demonstrated that exogenous protein HPL (Fib-H′) was successfully expressed in TBH (*Hpl*/*Hpl*) silkworms.

Interestingly, in the BmPSG there were almost no differences at mRNA and protein level between TBH and wild-type (WT) (−/−) in two components of endogenous fibroin, the Fib-L and P25 (Fig. 2A–C). However, mRNA levels of another fibroin component, Fib-H, were significantly down-regulated (Fig. 2A,B), and expression levels of Fib-H protein were also significantly reduced (Fig. 2C). Using the Fib-L protein as an internal control, the ratio of scanning values of Fib-H protein spots in mutant TBH and WT was 0.62. These results indicated that the expression of *Hpl* (Fib-H′) in the BmPSG of the mutant only reduced the synthesis of Fib-H (Table 1).

The protein composition of secreted cocoon silk was further quantitatively analyzed. In the composition of fibroin, the molar ratio is Fib-H (350 kDa): Fib-L (26 kDa): P25 (30 kDa) (n: n: n) = 6: 6: 1^14^,^44^. Fig. 2C results show that the molar ratio of Fib-H between TBH and WT was 0.62. Using the Fib-L protein as an internal control, the ratio of spots scanning values was HPL: Fib-L = 2.215: 1 in mutant TBH cocoon silk proteins, and the molar ratio was HPL: Fib-L = 0.48: 1 based on their molecular weight. While the ratio of spots scanning values was HPL: Fib-L = 0: 1 in WT cocoon. Therefore, HPL content in mutant TBH fibroin was 18.85%, which was calculated according to Fig. 2C and Table 1.

Earlier studies have shown that three fibroin proteins, Fib-H, Fib-L, and P25 were regulated mainly at the transcriptional level in the BmPSG^14^,^45^,^46^,^47^,^48^. Interestingly, in the TBH system obtained in this experiment, mRNA transcriptional levels of *Fib-H* and *Fib-L* were 1375 and 1358 times that of the foreign *Hpl* gene (Fig. 2B), but HPL protein accounted for 18.85% of the total amount of secreted fibroin (Fig. 2C).This suggested that the efficiency of posttranscriptional translation of the foreign *Hpl* gene, and secretion of the foreign protein, increased significantly, suggesting that the low transcriptional level and high translational level of exogenous protein HPL appeared in the silk gland cells of the TBH system. This result implied that changes in the regulation mechanism of protein synthesis occurred in the silk gland cells of TBH.

### Development and morphology of the TBH silk gland

**Figure 3:**
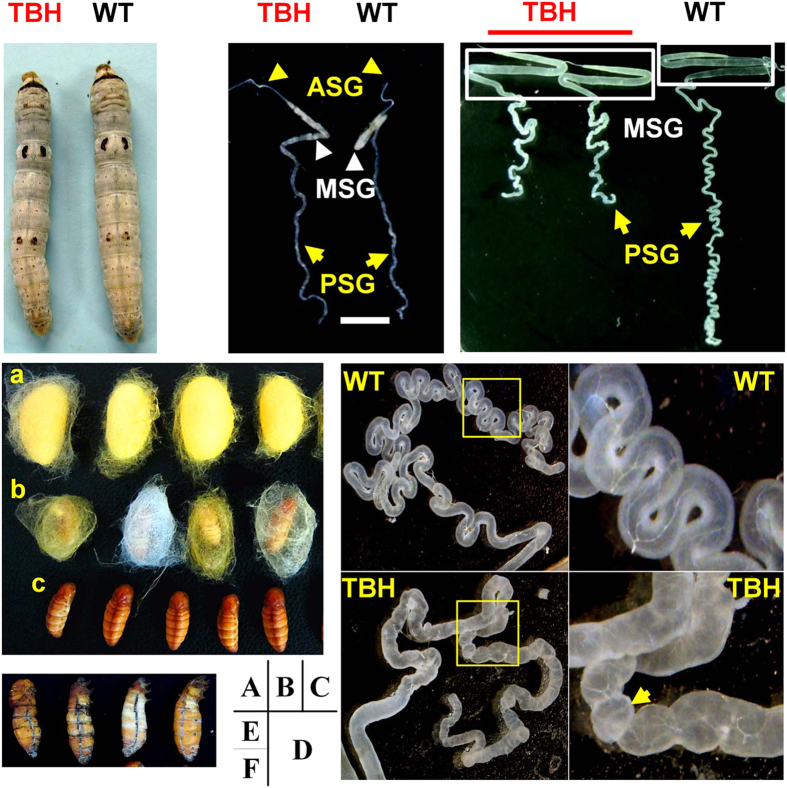
Developmental defects in TBH. (**A**) The 5th instar larvae. (**B**) The BmSG of 4th instar larvae. (**C)** and (**D**) The BmSG of late 5th instar larvae. (**E**) Cocooning status. a, normal cocoons. b, thin-shelled cocoons due to reduced spinning. c, naked pupae that did not spin. (**F**) Half pupated pupae. Bars are 10 mm in A, B, C, D and E, and are 1mm in F.

**Figure 4:**
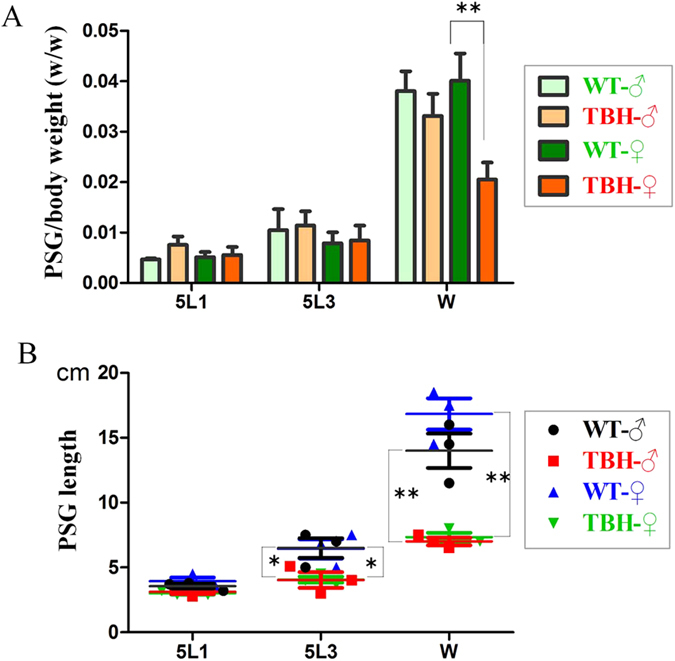
The weight ratio of the posterior silk gland (BmPSG) to animal body (A) and the length of BmPSG (B). *P < 0.05 and **P < 0.01.

Morphological observations showed there were no significant differences in the bodies of 5th instar larvae (Fig. 3A) and silk gland developmental status of the 4th instar larvae (Fig. 3B) between TBH (*Hpl*/*Hpl*) and WT (−/−). At late 5th instar, larvae whose silk glands grew to maximum size were observed. Although the growth and development of the MSG and the ASG in TBH were normal, the BmPSG in TBH showed significant differences from the WT (Fig. 3C and D). The BmPSG in WT were slender and more folded, while in TBH they became stubby and less folded (Fig. 3C). The folding and bending numbers of the BmPSG were only 33.4% ± 0.72% of those in the WT. The weights (Fig. 4A) and lengths (Fig. 4B) of the BmPSG in TBH were also significantly less than the WT controls. The BmPSG in WT had smooth surfaces and good translucence, while the BmPSG in TBH had nodules arranged in beadlike chains and were brittle, with poor tissue resilience and poor translucence (Fig. 3D). Some TBH mature larvae had spinning dysfunctions, resulting in thin-shelled cocoons and naked pupae, due to significant reductions in spinning amounts (Fig. 3E). Sometimes half pupated pupae appeared because of abnormal pupation (Fig. 3D).

**Figure 5:**
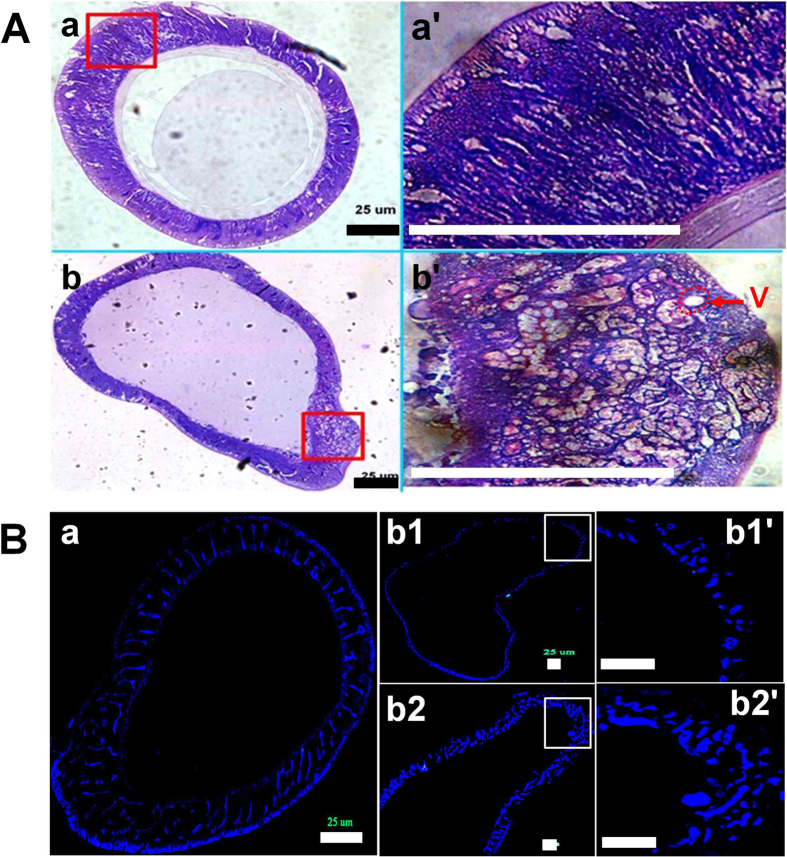
Hematoxylin and Eosin (HE) (A), and DAPI (B) staining of cross-sections of the BmPSG of mature larvae. a, wild type. The nuclei stained by HE and DAPI were branched and arranged in a compact and orderly manner. b, TBH nuclei were disordered. The b1 and b2 are different TBHs. Arrow represents the vacuole. Bars are 25 μm.

The BmPSGs of late 5th instar larvae were sectioned and observed. The results showed that the nuclei of the BmPSG cells in WT were extremely branched and arranged in a compact and orderly manner, and they spread within the entire cytoplasm (Fig. 5Aa,Ba). Nuclei of the BmPSG of some TBH individuals were less branched (Fig. 5Ab,Bb) and disorderly, with increased vacuoles (Fig. 5Ab).

The above results showed that during the process of synthesizing exogenous protein HPLs in the BmPSG cells of TBH, the nuclear morphology of cells with characteristics of efficient synthesis and secretion of silk proteins were affected, the growth and development of the BmPSG experienced significant changes, and subsequent pupation and metamorphosis of the organisms were also affected, which further suggested that changes in regulatory mechanisms of protein synthesis may have occurred in the silk gland cells of TBH.

### Comparative transcriptional profiling analysis

Ma *et al.*^49^ reported that the expression of a *Ras1CA* gene in the BmPSG of silk worm resulted in significantly enlarged silk gland cells. Protein synthesis and cocoon silk production increased by 60%, while feed consumption increased by only 20%, which indicated that, in addition to silk protein coding genes, other important functional genes like silk gland cell growth genes also affected protein synthesis and cocoon silk production in the silk gland cells.

It is known that the piggyBac transposon allowed foreign genes to be randomly inserted into the host genome. To confirm whether the BmPSG malformations in TBH mutants were caused by gene mutation in BmPSG as a result of the insertion destroying the normal developmentally-related functional gene, so the insertion site sequences were cloned, using piggyBac left arm primers and right arm primers (see Supplementary Table S2 online), based on the method of Thomas *et al.*^50^. The results showed that there were three insertion sites of exogenous TBH genes on chromosome 7 (nscaf2981: 91665..91665), chromosome 16 (nscaf3058: 1571815..1571815), and chromosome 28 (nscaf3099: 2236857..2236857), but none of these three insertion sites were within functional gene sequences. The morphology of the BmPSG, individual growth and bioecology characters were exactly all the same among TBH silkworms of the three different insertion sites, and no reports were found that the random insertion of piggyBac transposon in silkworm caused the similar phenotype of TBH silkworm (Table 2). Therefore, we believe that although *Fib-H*′ insertion and expression in TBH did not result in direct mutations of functional genes in the genome, insertion could have led to changes of genomic expression in the BmPSG cells.

**Figure 6:**
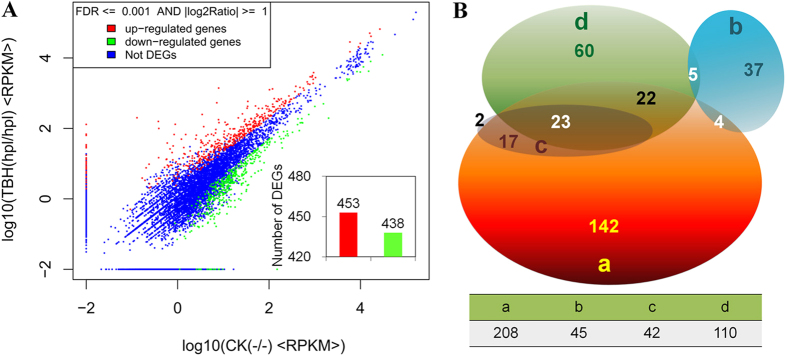
Differential genes and functional classification from DEGs. (**A**) Gene expression level of WT vs TBH. The 453 up-regulated genes are shown in red, and 438 down-regulated genes in green (insert, bottom right). DEGs (891) were genes with FDR ≤ 0.001 and fold difference of less than 2. (**B**) Pathways (Q value ≤ 0.05) were significantly enriched from 312 DEGs. a, Protein Metabolism; b, Protein Processing and Export; c, Energy Supply to Cells; d, Stress and Repair. TBH (*Hpl*/*Hpl*) was the mutant system for which the BmPSG of 5th instar larvae specifically expressed the *Hpl* gene (Fig. 2). The most typical biological phenotype was the retardation of the BmPSG of late 5th instar larvae (Fig. 3), so we chose the BmPSG of the wandering stage silkworm for DEGs analysis, which are about to spin.

RNA-seq DEG analysis showed that a total of 891 differentially expressed genes, including 453 up-regulated and 438 down-regulated genes, were detected in the BmPSG tissues of TBH and WT silkworms (Fig. 6A), among which 656 genes were annotated. Pathway enrichment analysis of these 656 annotated genes was further carried out, and 232 related pathways were obtained. The 312 annotated genes participated in 31 pathways closely linked to phenotypes of TBH, and DEGs degree of enrichment reached 47.6% (see Supplementary Table S3 online, Fig. 6B). Gene ontology based functional classification (see Supplementary Fig S1 online) and statistics (see Supplementary Table S3 online) showed that the 312 annotated genes were involved in four types of pathways, of which 208 genes were classified as involved in protein metabolism, accounting for 31.7% of the total number of annotated genes. Another 110 annotated genes were grouped as stress and apoptosis genes, accounting for 16.8%, 42 annotated genes participated in energy supply to cells, accounting for 6.4%, and 46 annotated genes were related to protein processing and export, accounting for 7% (Fig. 6B). Nine differentially expressed genes were sampled for verification by transcriptional level experiments, and the results were consistent with those of DEG analyses (see Supplementary Fig. S2 online), indicating that the results of DEG were reliable.

The KEGG pathways associated with DEG were further ranked by degree of difference in significance. Table 2 lists the top 10 pathways that had the greatest impact on the BmPSG cells of TBH (*Hpl*/*Hpl*) mutants. The first pathway was “Protein processing in the endoplasmic reticulum”. As is well-known, the fibroin belongs to secreted proteins and firstly need the endoplasmic reticulum of processing after the fibroin is synthesized. So the anomaly of this process was in accord with the phenotype of TBH, which the powerful ability of synthesising and secreting proteins was significantly attenuated in the BmPSG. In addition, the fifth pathway was “protein export”. The anomaly of this process also implies the direct effect to the process of the fibroin secretion. The results in Supplementary Table S3 online and Fig. 6B also show that expression of genes related to stress and apoptosis are significantly affected. The cellular stress metabolism in the BmPSG of TBH (*Hpl*/*Hpl*) was enhanced and ROS levels increased sharply (see Supplementary Fig. S3 online), indicating that changes in damage and repair functions in the BmPSG cells of the mutants were affected. It is worth noting that the 10 pathways with greatest impact included pathways related to Parkinson’s disease, Huntington’s disease, and Alzheimer’s disease, which include many genes related to synuclein and neurotransmitter, indicating that neurobehavior in silk gland tissues and cells of the mutants was also affected. These results suggested that metabolism and neurobehavior control may affect the development of silk glands in TBH, and cause retarded gland development. The mechanism may therefore be associated with oxidative stress and metabolic repair in dysfunctional silk gland cells.

## Discussion

The BmPSG cells of silkworms can efficiently synthesize three components of fibroin, Fib-H, Fib-L, and P25. Low molecular weight proteins Fib-L and P25 can be directly secreted into the gland lumen in monomeric form, while macromolecules such as Fib-H can only be transported and secreted, forming a complex with Fib-L^45^,^51^. Many studies (see Supplementary Table S1 online) have shown that exogenous small molecular proteins such as GFP, DsRed, and bioactive peptide could be synthesized in the silk gland cells and secreted directly into the gland lumen and outside of the cell body, but the synthesis efficiency in the silk gland cells was not high. When expression of Fib-H in the BmPSG cells was knocked out, the expression and secretion of Fib-L and P25 were almost completely inhibited^42^. Though EGFP was high level expressed and secreted in PSG which Fib-H gene was knocked out by Ma *et al.*^44^, but in our experiment, the HPL is a macromolecular protein, which molecular weight is much greater than that of EGFP. The regulation of the secretion of HPL and EGFP after their synthesis in the silk gland cells would not be the same. So the ratio 18.85% of HPL in TBH fibroin was a breakthrough in the field of expressing a macromolecular protein in silk gland.

In the BmPSG cells, normal expression levels and the relative proportions of three endogenous fibroin protein genes *Fib-H*, *Fib-L*, and *P25* did not almost change in existing BmPSG exogenous gene expression platforms (see Supplementary Table S1 online). In the BmPSG cells of transgenic TBH mutants, levels of transcripts, and protein synthesis and secretion of Fib-L and P25 did not decline. However, the transcripts, and protein synthesis and secretion of Fib-H were down-regulated, therefore high level of translation and secretion of exogenous macromolecular protein HPL was achieved at low levels of transcription. This result implied that construction of a stable knockdown system of Fib-H, or other fibroin and sericin proteins, would be an effective way to achieve high efficiency in expression and secretion of exogenous proteins in the BmSG.

Although exogenous protein HPL was successfully expressed and efficiently secreted in the BmPSG, the BmPSGs of some TBH mutants appeared developmentally abnormal, and this directly affected the metamorphosis from larvae to pupae. The total amount of exocrine silk proteins in the mutants decreased, and no spinning appeared in some individuals (Fig. 2). DEG analysis in the BmPSG cells further showed that the expression of 208 genes related to protein metabolism, gene expression associated with stress and apoptosis, and other neural responses were also significantly affected in the BmPSG cells of the transgenic TBH mutant, suggesting that BmPSG retardation in TBH may be caused by different mechanisms, such as metabolism and behavior control, and the mechanism may also be associated with oxidative stress and metabolic repair in dysfunctional silk gland cells.

In conclusion, these studies provide a guiding significance of silk glands as a system for recombinant protein production. The results showed that 1) reduced expression of endogenous silk protein genes in the silk gland cells effectively improved the efficiency of expression of exogenous proteins, and 2) expressing exogenous proteins in the BmSG cells may have caused reprogramming of genome expression. Therefore, editing of a single target gene could initiate changes in expression of a wide range of functional genes other than the target gene, and may have a negative impact on BmSG tissues and cells, as well as animal bodies, which could be lethal. Therefore, in order to investigate methods to improve the expression efficiency of exogenous proteins in silk glands, future studies to balance the relationship between yield and vitality should be done. As with animal breeding, further studies should be conducted to determine the impact of genome reprogramming on host vitality.

## Materials and Methods

### Designing and construction of the *Fib-H*′ gene

We designed a coding sequence *Hpl* with the full-length 3057 bp (see Supplementary sequence online). *Hpl* contained a large number of repeats and 87.53% coding sequences for Ala, Ser, and Gly. The encoded HPL protein had 11 Cys residues which could enhance the binding of HPL with Fib-L protein synthesized in the silk glands. The *Hpl* sequence was synthesized by BGI (BGI, Shenzhen, China). The *Hpl* was spliced into the *Fib-H* cloned from the silk worm Dazao strain, between 2311 bp of the 5′ end sequence (AF226688.1, 79027-79359, GenBank) and 333 bp of the 3′ end sequence (GenBank AF226688.1, 61543-62437); thus, a new gene (*Fib-H*′) of 5701 bp length was constructed. *Fib-H*′ was then inserted into plasmid pSLfa1180fa (3.5 kb in size), and was further cloned into the AscI site of pBac [3xp3-DsRedaf] to construct a recombinant vector pBac [3xP3-DsRedaf] -*Fib-H*-*Hpl*, for embryo injection.

### Silkworm embryo microinjection

Vector pBac [3xP3-DsRedaf]-*Fib-H*-*Hpl* was purified with the QIAGEN Plasmid Midi kit (Qiagen K.K., Tokyo, Japan). A non-autonomous plasmid, pHA3PIG, was used as the helper for the production of the transposase. Embryos injections were performed as described by Wang^42^. After injection, the eggs were incubated in a sterile environment at 25 °C. The mating of male and female G0 moths was random. The GM pupae were separated at G1, by visualizing under fluorescence microscopy (Olympus SZX16, Osaka, Japan). Successive selection was until G6, then after stabilization at GM, pupae were used for further studies. All embryos used strain N4w.

### Tissue staining

The levels of ROS were measured using the ROS kit S0033 (Beyotime, Nantong, China) and followed the method of Liu *et al.*^52^. The posterior BmSG (BmPSG) were collected in DEPC (containing 0.7% NaCl) to avoid air exposure and then washed three times in normal saline. The BmPSG were then quickly placed into the staining solution for 15 min, and then washed for 5 min with saline, in the dark. The green fluorescence from ROS was observed at an excitation wavelength of 488 nm and emission wavelength of 525 nm, with a fluorescence microscope (Olympus SZX16, Tokyo, Japan).

The BmPSG technology and ultrastructure of *Bombyx* larvae were observed using light microscopy, after hematoxylin and eosin (HE) staining. The nuclei of the dividing and damaged BmPSG cells were observed using fluorescence microscopy after staining with 4′,6-diamidino-2-phenylindole (DAPI). HE and DAPI staining followed the method of Ji *et al.*^39^. BmPSG were paraffin imbedded and sectioned at a thickness of up to 5–10 μm, then the sections were dewaxed using xylene, and rehydrated in an ethanol series. Sections were stained with HE or with DAPI. DAPI and HE were purchased from Invitrogen (Carlsbad, CA, USA).

### Gene expression analysis

Total RNA was isolated from the BmPSG at the wandering stage using sqRT-PCR RNAiso Plus (TaKaRa, Dalian, China). The cDNA was synthesized using the Perfect Real Time version of the PrimerScript™ RT reagent kit with gDNA Eraser (Perfect Real Time) (TaKaRa, Dalian, China) according to the manufacturer’s instructions. Then, sqRT-PCR and qRT-PCR were selected to analyze the genes. Using ABI Stepone Plus (Ambion, Foster City, CA, USA) and the fluorescent dye SYBR Premix Ex Taq (TaKaRa), qRT-PCR was performed in a total reaction volume of 20 μl, according to the manufacturers’ instructions. The *Bombyx* NRp49 gene was selected as the internal control. Primers used in this study are listed in Supplementary Table S2 online.

### Analysis of the cocoon proteins

SDS-PAGE and western blotting were used to identify HPL protein in cocoon silk. Proteins were extracted from 35 mg cocoon shells, which were dissolved in 1 ml 9 M LiSCN for 2 hours, followed by centrifugation for 15 min at 10000 rpm. The protein concentrations of extracts were determined by the BCA Protein Assay Kit (Beyotime, Nantong, China) using a microplate reader (EonC™ BioTek™; Fisher Scientific, Waltham, MA, USA).

One hundred μg total proteins from cocoon shells were subjected to SDS-PAGE. The extracts were separated for 30 min on an 8% running gel at 80 V, and then further electrophoresed for 80 min on a 5% stacking gel at 120 V. Gels were visualized by silver staining and analyzed densitometrically using the Gel-PRO ANALYZER software.

Western blotting used polyclonal antisera against the HPL protein. After peptide sequence design, synthesis, and purification, a peptide sequence from the HPL protein, NH_2_-VEKSKHLYEEKKSEC-CONH_2_, was used as an antigen to immunize New Zealand rabbits. The antibody production was conducted by Abgent Biotechnology Co., Ltd (Suzhou, China). The samples were separated on 10% SDS-PAGE gels, and then transferred to polyvinylidene difluoride membranes (PVDF). The membranes were blocked with a blocking solution, followed by incubation with anti-HPL antibody, and then washed and incubated with horseradish peroxidase (HRP)-labeled anti-rabbit IgG (Bioworld Technology, Minneapolis, MN, USA). Proteins were visualized using the EZ-ECL Chemiluminescence Detection Kit for HRP (Biological Industries, Beit Haemek, Israel).

### RNA-Seq

For Illumina sequencing, equivalent quantities of total RNA were isolated from the three larvae and BmPSG were pooled. After poly (A) mRNA was purified and fragmented into smaller fragments, random hexamer primers and reverse transcriptase (Invitrogen, Life Technologies, Carlsbad, CA, USA) were used to carry out first strand cDNA synthesis. Second strand cDNA synthesis was performed with RNase H (Invitrogen) and DNA polymerase I (New England BioLabs, Beijing, China). We constructed a cDNA library with average insert sizes of 200–500 bp and conducted cDNA sequencing using the Illumina HiSeq™ 2000 system according to the manufacturer’s protocols, with a read length of 50 bp. RNA-Seq Quantification analyses used two independent cDNA libraries, and were constructed for the two organs in parallel according to the RNA-Seq protocol. The RNA-seq sequencing data were made available to BGI (BGI, Shenzhen, China).

## Additional Information

**How to cite this article**: Wang, H. *et al.* High yield exogenous protein HPL production in the *Bombyx mori* silk gland provides novel insight into recombinant expression systems. *Sci. Rep.* **5**, 13839; doi: 10.1038/srep13839 (2015).

## Supplementary Material

Supplementary Information

## Figures and Tables

**Table 1:** Relative percent analyses of different protein bands.

**Protein production**	**TBH(*Hpl*/*Hpl*)**	**WT(−/−)**
Fib-H/fibroin (w/w) (%)	71.01	91.86
HPL/fibroin (w/w) (%)	18.85	0
HPL/cocoon shell (w/w) (%)	15.46	0

The silk fibroin was approximately 82% of the cocoon shell silk protein in the experimental varietiy, and the molar ratio of fibroin is Fib-H (350 kDa) : Fib-L (26 kDa) : P25 (30 kDa) (w:w:w) = 6:6:1^14^,^44^. The molecular mass of HPL protein was approximately 120 kDa. Using Fib-L as an internal control, HPL radio of fibroin in TBH was calculated according to the following formula: HPL(%) = W(HPL) ÷ [W(HPL) + W(Fib − H) + W(Fib − L) + W(P25) × 100]. In the formula,  = molecular weight (M)×mole number (n). SDS-PAGE bands were quantified by a Gel-PRO ANALYZER.

**Table 2:** The greatest functional classification differences between the wild type and mutant pathways.

**Arrangement**	**Pathway**	DEGs withPathwayannotation (656)	**All genes withpathway annotation(9056)**	**P value**	PathwayID
1	Protein processing in endoplasmic reticulum	40 (6.1%)	180 (1.99%)	9.115041e-11	ko04141
2	Oxidative phosphorylation	27 (4.12%)	120 (1.33%)	8.26374e-08	ko00190
3	Parkinson’s disease	23 (3.51%)	119 (1.31%)	1.168411e-05	ko05012
4	Proteasome	12 (1.83%)	40 (0.44%)	1.596926e-05	ko03050
5	Protein export	8 (1.22%)	20 (0.22%)	4.182822e-05	ko03060
6	Huntington’s disease	31 (4.73%)	221 (2.44%)	0.0002724467	ko05016
7	Metabolic pathways	140 (21.34%)	1483 (16.38%)	0.0003256242	ko01100
8	Alzheimer’s disease	26 (3.96%)	177 (1.95%)	0.0004059375	ko05010
9	Citrate cycle (TCA cycle)	11 (1.68%)	52 (0.57%)	0.001033604	ko00020
10	Antigen processing and presentation	8 (1.22%)	34 (0.38%)	0.002436458	ko04612
