# Supplementary material for: High yield exogenous protein HPL production in the Bombyx mori silk gland provides novel insight into recombinant expression systems
Source: Sci Rep. 2015 Sep 15;5:13839. doi: 10.1038/srep13839 (PMC4570194; doi:10.1038/srep13839)
Supplement: Supplementary Information [file srep13839-s1.doc]

Supplementary Information

**High yield exogenous protein** **HPL production in the *Bombyx mori* silk gland provides novel insight into recombinant expression systems**

Huan Wang^1,🞤^, Lu Wang^1,🞤^, Yulong Wang^1^, Hui Tao^1^, Weimin Yin^1^, Yanghu SiMa^1^, Yujun Wang^2,🞸^, Shiqing Xu^1,3,🞸^

1 School of Biology and Basic Medical Sciences, Medical College, Soochow University, Suzhou 215123, China.

2 R&D Division, Okamoto Corporation, Nara 635-8550, Japan.

3 National Engineering Laboratory for Modern Silk, Soochow University, Suzhou 215123, China.

^🞸^ Corresponding author: szsqxu@suda.edu.cn (S. Xu), wangxiaochen528@hotmail.com (Y. Wang) ^🞤^ These authors contributed equally to this work.

**Table of Contents:**

Fig. S1. Gene ontology analysis.

Fig. S2. RNA-Seq result verification

Fig. S3. Detection of reactive oxygen species (ROS) in posterior silk gland (BmPSG).

Table S1. The level of recombinant proteins expressed in silk gland of transgenic silkworm.

Table S2. The gene-specific primers used in this study.

Table S3. Classification of DEG enriched in pathways.

Supplementary sequence. The nucleotide sequence of *Hpl* and its deduced amino acid residues.

**Supplementary Figures**


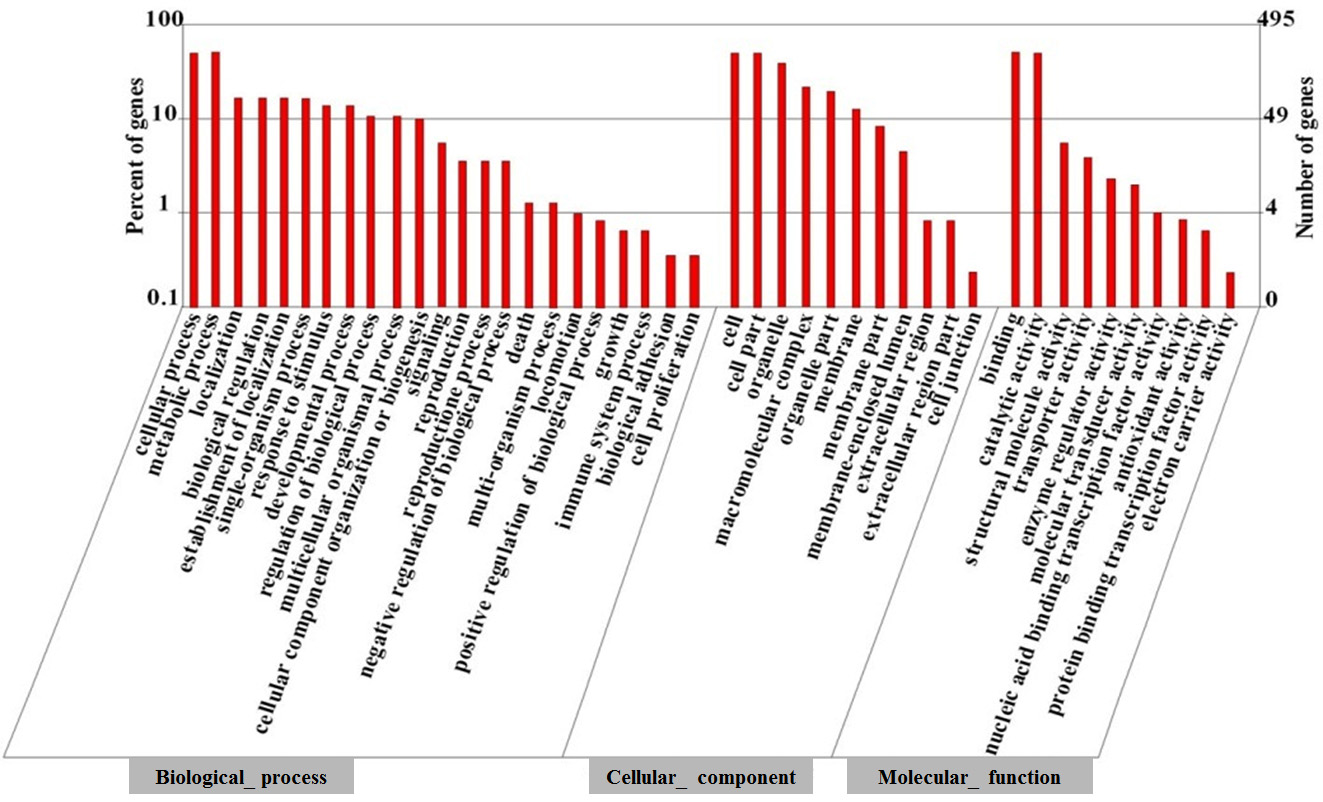


**Fig. S1 Gene ontology analysis.** GO annotation information of 656 differentially expressed genes was obtained using Blast2 GO software. The statistical analysis of GO functional classifications was conducted using WEGO software ^[53]^ to cognize the features of functional distribution of differentially expressed genes from a macroscopic aspect.

**
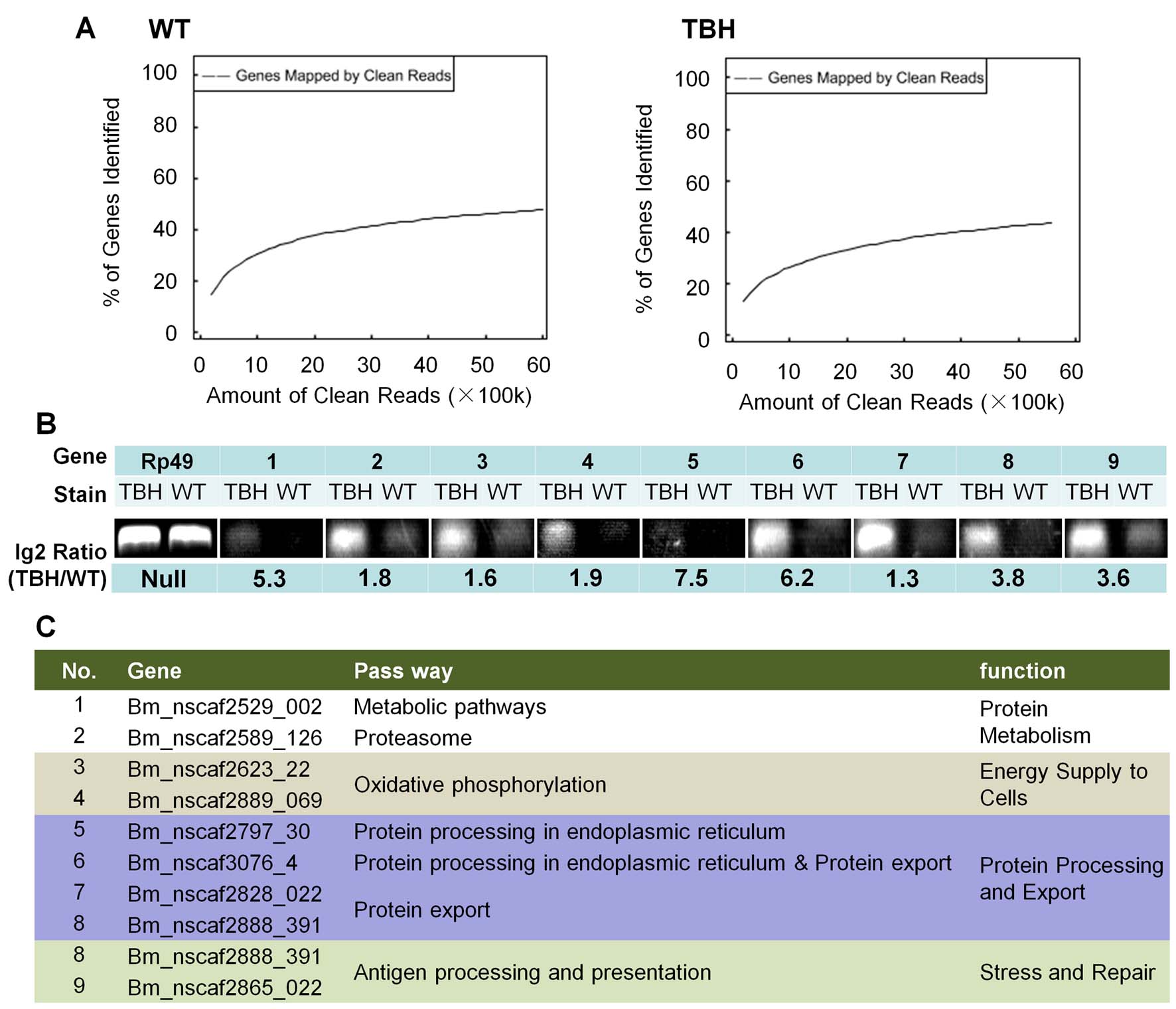
**

**Fig. S2 RNA-Seq result verification.** (A) Saturation analysis. The number of detected genes became more highly saturated and showed that the materials were a qualified database. (B) RT-PCR verification. The Rp49 gene was selected as an internal control. The gene names and functions are described in graph (C).

**
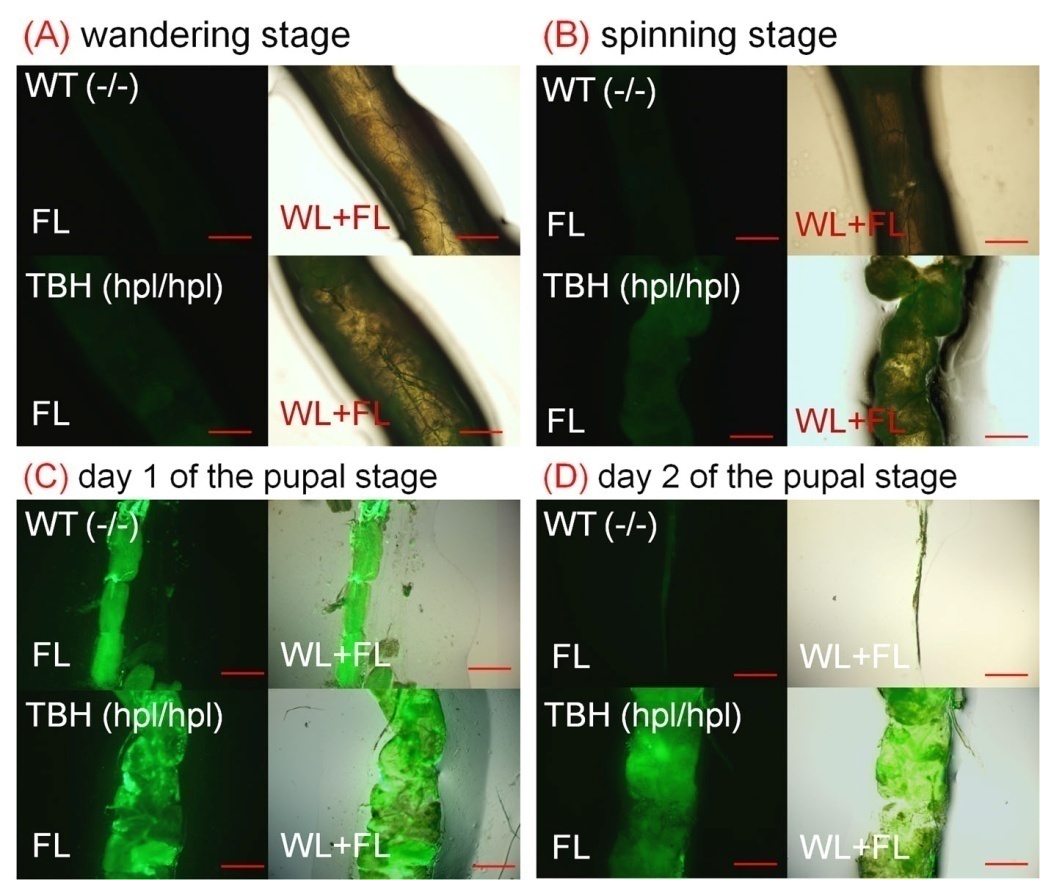
**

**Fig. S3 Detection of reactive oxygen species (ROS) in posterior silk gland (BmPSG).** (A) At the wandering stage, the BmSG developed to the maximum, and trace ROS were produced in part of the BmPSG of TBH; (B) At the spinning stage, ROS levels in the BmPSG of TBH were significantly higher than that of WT, exhibiting a very high stress state. (C) On day 1 of the pupal stage, apoptosis, and autophagy of degenerative processes began in the BmSG, and high levels of ROS existed in the BmPSGs of both WT and TBH. (D) On day 2 of pupal stage, the BmPSG of WT degenerated to the end, BmPSG of TBH was still in the middle of degeneration, and a high level of ROS was still maintained in tissues. WL, visible light. FL, fluorescence. Excitation wavelength was 488 nm and emission wavelength was 525 nm. Bar, 100 μm.

**Supplementary Tables**

**Table S1 The level of recombinant proteins expressed in** silk gland of transgenic silkworm

| **Year** | **First Author** | **Exogenous protein** | **Silkworm strain** | **Promoter** | **Expression level of procucts** |
| --- | --- | --- | --- | --- | --- |
| **2014** | Wang F^[24]^ | human acidic fibroblast growth factor (hFGF1) protein | Dazao | Ser1 | approximate 0.07% of the cocoon shell weight |
| **2014** | Xu H^[25]^ | Infectious bursal disease virus (IBDV) capsid protein VP2 | D9L | Ser1 | a yield of 3.33 mg (purity > 90%) purified from 30g cocoon |
| **2014** | Kuwana Y^[26]^ | Spider dragline silk | C515 | Fib-H | 0.37 to 0.61% (w/w) native silkworm fibroin |
| **2014** | Sato M^[27]^ | Single-chain variable fragment | pnd-w1 | Fib-L | 5%-10% of the total Fib-L |
| **2014** | Song Z^[28]^ | Human insulin-like growth factor | Dazao×Haoyue | Sericin-1 | approx 162.7 ng/g of cocoon shell weight |
| **2014** | Li Z^[29]^ | antibacterial silk | GaoBai | Fib-L | null |
| **2013** | Wang F^[30]^ | DsRed | Dazao | Ser1 | 9.5 % (w/w) of cocoon shell weight |
| **2013** | Iizuka T^[31]^ | EGFP, DsRed &Monomeric Kusabira orange | pnd-w1 | Fib-H | null |
| **2012** | Xue R^[32]^ | Human granucyto-macrophage | GaoBai | Fib-H | approx 2.70 ng/g of freeze dried posterior silk gland |
| **2012** | Teulé F^[33]^ | Partial spider silk fibroin sequence | pnd-w1 | Fib-H | approx 2%-5% (w/w) of cocoon shell weight |
| **2011** | Li Y^[34]^ | Human insulin-like growth factor | GaoBai | Fhx | approx 150 ng/g of fresh posterior silk gland |
| **2011** | Nagano A^[35]^ | Ca binding sequence | pnd-w1 | Fib-H | Null |
| **2010** | Nakazawa Y^[36]^ | Partial fibronectin sequence &Partial collagen sequence | pnd-w1 | Fib-L | Null |
| **2010** | Zhao A^[37]^ | EGFP | N4 | Fib-H | 15% (w/w) of silk fibroin |
| **2010** | Adachi T^[16]^ | Non-triple helical collagen α chain | pnd-w1 | Ser1 | 8% of the dried cocoon silk |
| **2010** | Tatematsu K^[22]^ | EGFP | pnd-w1-Nd -Sd pnd-w1 | Ser1 | 500 μg per larva |
| **2010** | Zhu Z^[38]^ | Partial spider silk fibroin sequence | pnd-w1 | Fib-H | 5% (w/w) of silk fibroin |
| **2009** | Iizuka M^[39]^ | Mouse monoclonal antibody | pnd-w1 | Ser1 | 1.1% of the dried cocoon silk |
| **2009** | Tateno M^[17]^ | Human μ-opioid receptor | pnd-w1 | Fib-L | 150-250 ng/g of fresh silk gland |
| **2009** | Zhao Y^[18]^ | Human insulin-like growth factor-I | Haoyue | Ser1 | 2.44 ng/g of middle silk gland |
| **2007** | Kurihara H^[11]^ | Feline interferon | pnd-w1 | Fib-H | 6% of the dried cocoon silk |
| **2007** | Ogawa S^[19]^ | Human serum albumin | pnd-w1 | Ser1 | 0.3% of the dried cocoon silk |
| **2007** | Yanagisawa S^[13]^ | Collagen & Fibronectin derived peptides | pnd-w1 | Fib-L | 0.2% & 0.6% of the dried cocoon silk |
| **2006** | Adachi T^[15]^ | Mini human collagen & rolyl-hydroxylase α-subunits | pnd-w1 | Fib-L | Null |
| **2006** | Hino R^[20]^ | Human basic fibroblast growth factor | pnd-w1 | Fib-L | 0.04% of the fibroin |
| **2005** | Inoue S^[21]^ | Fib-L-GFP | C108 & Nd-Sd | Fib-L | 10% of the dried cocoon silk |
| **2003** | Tomita M^[9]^ | Human type III procollagen | pnd-w1 | Fib-L | 0.8% of the dried cocoon silk |

**Table S2** The gene-specific primers used in this study

| Functions | Primers | Sequences |
| --- | --- | --- |
| Sites of transposon-insertion mutant | pBacL LA | S-ATCAGTGACACTTACCGCATTGACA  A-TGACGAGCTTGTTGGTGAGGATTCT |
|  | pBacL RA | S-TACGCATGATTATCTTTAACGTA  A-GGGGTCCGTCAAAACAAAACATC |
| sqRT-PCR | Bm_nscaf2889_069 | S-CGAGGAATGTTTCAAGCGTTTT |
|  |  | A- TATGGGTGGTCTTTTGATGGGA |
|  | Bm_nscaf2828_022 | S- CCATTAGCAAAAGGAAGCC |
|  |  | A- TTACCAAGGAACGACCCGA |
|  | Bm_nscaf2865_022 | S-CGACCCTGCCAATAAAGA |
|  |  | A- CACCGAAAGCCCAACAAC |
|  | Bm_nscaf2888_391 | S- CCTCCTGCTCCTCGTGGT |
|  |  | A- CCTGTTTCCTGTTCCCTT |
|  | Bm_nscaf3076_4 | S- GTGCAGAATTTCACCCCA |
|  |  | A- GACTCCTCGTCCAGCCGT |
|  | Bm_nscaf2529_002 | S- CCTTACGACAACCCAAAC |
|  |  | A- GTGAATAAAATCCCGACC |
|  | Bm_nscaf2589_126 | S-AGCCATCACAGTATTTTCTCCT |
|  |  | A- CATCCACTCCTCTAACTCCAAC |
|  | Bm_nscaf2623_22 | S-CTGAATGGTTTGGTTGGCTA |
|  |  | A-AAAGATTTTCGGTGTGGTCC |
|  | Bm_nscaf2797_30 | S- TTTCATTCCGTTCTCCAT |
|  |  | A- GTCGTCTCGCTGCTTGTT |
| sqRT-PCR or qRT-PCR | Fib-H | A-ACAAGGTGCAGGAAGTGC |
|  |  | S- AGCAATTCACACAAGGCAGT |
|  | Fib-L | S- CCGGAGGTGGAAGAATCTAT |
|  |  | A- GGTTATGTAGGCAGCGATGT |
|  | P25 | S- CCCTGCTACTTGGACGATT |
|  |  | A- GATTATGGTCGACGTAGGTG |
|  | *Hpl* | S- ATGGACCCAGGGAGCACAAG |
|  |  | A- AGACAATAATTTATTTCATTTATAA |
|  | Rp49 | S- GCATCAATCGGATCGCTATG |
|  |  | A- GGACCTTACGGAATCCATTTG |

qRT-PCR, quantitative real-time reverse-transcription PCR; sqRT-PCR, semiquantitative reverse-transcription PCR.

**Table S3 Classification of DE**G enriched in pathways

| **Functional category (****DEGs)** | **Pathway** | **P value** |
| --- | --- | --- |
| **Protein Metabolism**  **（208）** | Proteasome | 1.596926e-05 |
|  | Metabolic pathways;  RNA transport;  One carbon pool by folate;  Alanine, aspartate and glutamate metabolism;  Tyrosine metabolism;  Ribosome;  Phenylalanine metabolism;  Ribosome biogenesis in eukaryotes;  Phenylalanine, tyrosine and tryptophan biosynthesis; Glycine, serine and threonine metabolism;  Ubiquitin mediated proteolysis; | 0.0003256242  0.005753618  0.01520213  0.02320089  0.05406263  0.0854069  0.09564275  0.1528777  0.2598001  0.5828363  0.8118458 |
| **Protein Processing and Export**  **（46）** | Protein processing in endoplasmic reticulum;  Protein export | 9.115041e-11  4.182822e-05 |
| **Energy Supply to Cells**  **（42）** | Oxidative phosphorylation;  Citrate cycle (TCA cycle);  Nicotinate and nicotinamide metabolism； | 8.26374e-08  0.001033604  0.00929588 |
| **Stress and Repair**  **（110）** | Parkinson's disease;  Huntington's disease;  Alzheimer's disease  Antigen processing and presentation;  Metabolism of xenobiotics by cytochromeP450;  Phagosome;  DNA replication;  Base excision repair;  Insect hormone biosynthesis;  Endocytosis;  Lysosome;  Regulation of autophagy;  mTOR signaling pathway;  MAPK signaling pathway; | 1.168411e-05  0.0002724467  0.0004059375  0.002436458  0.07215627  0.09345219  0.1262581  0.3514925  0.4556219  0.4563338  0.6265337  0.742013  0.8110317  0.9953146 |

**Supplementary sequence.** **The nucleotide sequence of *Hpl* and its deduced amino acid residues**

1 tataatagcggcgaatatggcggatggggatcgaacctagataggggctgcctcttcggctccgacgacggc

Y N S G E Y G G W G S N L D R G C L F G S D D G

73 ggaaggaagtcaaagcggtccagcgacgatagctcggccgccgccgctgccgccgccgccgcgagtggttca

G R K S K R S S D D S S A A A A A A A A A S G S

145 ggcggaggcgaaggttcggccgccgccgctgctgccgctgccgccgccagctcgggcggcagaagcagcgcg

G G G E G S A A A A A A A A A A S S G G R S S A

217 gccgcggccgccgccgcggcttcggccgcctctagtaaatacggtggtagcagcgctgctgccgccgccgct

A A A A A A A S A A S S K Y G G S S A A A A A A

289 gccgccgctgcttctggcggaggaaacggtggaggatgcgcagccgcggcagccgccgccgcagccgccgct

A A A A S G G G N G G G C A A A A A A A A A A A

361 gccggaaggtacggcagcagtagcgcagcagcagcagcagcagccgccggtagcggcggtggaggcggcggt

A G R Y G S S S A A A A A A A A G S G G G G G G

433 ggcggttcggccgcagccgcagcggcagccgcagcagcagccacgtctggaggaagaggaagaaactgtgcc

G G S A A A A A A A A A A A T S G G R G R N C A

505 gccgcagcagcagccgccgccgccgccgcttcggccggtggaggaggaagcgccgccgcggccgctgccgca

A A A A A A A A A A S A G G G G S A A A A A A A

577 gccgccgcttcgagcggatcaggaggcggagccgccgctgctgcggccgcagcagccgcgtcgggtggatcg

A A A S S G S G G G A A A A A A A A A A S G G S

649 ggaggcgcggccgcatccgccgctgccgcggcctcggccgcttccgaatctggaaaatccaaaagatctcat

G G A A A S A A A A A S A A S E S G K S K R S H

721 tcagatagtgcgtctgccgccgcagcagccgccagttccggaggatccggtagttccgctgcagctgctgcc

S D S A S A A A A A A S S G G S G S S A A A A A

793 gccgccgcagccgccggttccggaggatccggtagttccgctgcagctgctgccgccgccgcagccgccggt

A A A A A G S G G S G S S A A A A A A A A A A G

865 tccggaggatccggtagttccgctgcagctgcagccgccgcggccgcagccggcggttccggcggaagcgcc

S G G S G S S A A A A A A A A A A G G S G G S A

937 gctgccgctgccgctgctgcggccgcttcgggggcaaaatcaaaaagatcagaaggatcctgcaatagtgga

A A A A A A A A A S G A K S K R S E G S C N S G

1009 tccagtggaagttccggaagtagtagcgcggcagcagccgctgccgcggccgctagcggttccggaggcaag

S S G S S G S S S A A A A A A A A A S G S G G K

1081 ggaagcggaagttccgcagctgcagccgcagccgcggccgccagcggctccggaggcaagggaagcggaagc

G S G S S A A A A A A A A A S G S G G K G S G S

1153 tccgcagcggcagccgccgccgccgcagccagcggttccggaggcaagggaagcggaagctccgcagcggca

S A A A A A A A A A S G S G G K G S G S S A A A

1225 gccgcagccgcggccgccagcggttccggaggcaagggaggcggaagttctgcagctgcggccgccgccgct

A A A A A A S G S G G K G G G S S A A A A A A A

1297 gcagccagcggttccggaggtcatggtagcggaagctctgcatctgcagcggcagccgcggcggccagcggt

A A S G S G G H G S G S S A S A A A A A A A S G

1369 tccggaggcaaaggaagcggaagctcggcagctgcagccgccgccgccgcagccagcggctccggaggtcat

S G G K G S G S S A A A A A A A A A S G S G G H

1441 ggtggcggaagcagctcttcaggtgcctcttccgctgctagcggcagttccagcggtactactgcaaatgaa

G G G S S S S G A S S A A S G S S S G T T A N E

1513 aacaaccacaacgataatactagatgcaaaagatgcgtaggaaagagcggctatccaatggataataacgga

N N H N D N T R C K R C V G K S G Y P M D N N G

1585 tatccgtctaagtctgaaagtagcggtgcggcctcgtctgccgcaggctccgccagcggttcgggcagctcg

Y P S K S E S S G A A S S A A G S A S G S G S S

1657 gctgcttccgcagctgcagcagcagcagccagcggatccggaggcaaaggaggcggcagctccgcagcggca

A A S A A A A A A A S G S G G K G G G S S A A A

1729 gccgctgctgcggcagctagcggctccggaggcaagggcggaagctccgcagcggcagccgccgccgcggca

A A A A A A S G S G G K G G S S A A A A A A A A

1801 gccagcggttccggaggcggaagttccgcagcggcagctgccgcggcagccagcggttccggaggcggaagt

A S G S G G G S S A A A A A A A A S G S G G G S

1873 tctgcagcggcagccgccgcggccgccgctagcggttccggaggccgtggaggcggaagcagctcttcaggt

S A A A A A A A A A S G S G G R G G G S S S S G

1945 gcttcttccgcagctagcgggggttccagcggaactcaagataataaatgcaaaagatgcgcaggaaagagc

A S S A A S G G S S G T Q D N K C K R C A G K S

2017 agcaatgacggatacccatctgggtctggaggcagcggtacagctgcatcgtccggcgcgtcctctgccggt

S N D G Y P S G S G G S G T A A S S G A S S A G

2089 ggctcaggtggctctgccgcctccgcggccgcggccgcagcagcctcaggcggcaagggaggcggaagcgcc

G S G G S A A S A A A A A A A S G G K G G G S A

2161 gcagcggcagccgctgctgcggcagccagcggttccgggggcaagggaggcggaagctccgcagcggcagcc

A A A A A A A A A S G S G G K G G G S S A A A A

2233 gccgctgcagcagccagcggttccggaggcggaagttccgcagcggcagccgccgcggccgccgccagcggt

A A A A A S G S G G G S S A A A A A A A A A S G

2305 tccggaggccacggaggcggaagcagctcttcgggtgcttcttccgctgctagcgggggttccagcagctcc

S G G H G G G S S S S G A S S A A S G G S S S S

2377 agcggaattaaagataagaaatgcaaaagatgcgcaggaaagagcagcaataacggatacccatctgggtct

S G I K D K K C K R C A G K S S N N G Y P S G S

2449 ggaggcagcagtacagctacatcgtccggtgcgtcctctgccggtggttcaggtggctctgccgcctccgct

G G S S T A T S S G A S S A G G S G G S A A S A

2521 gccgcggccgcagcagccgcaggcggcaatggaggcggaagcgccgcagcggcagccgctgccgcggcagcc

A A A A A A A G G N G G G S A A A A A A A A A A

2593 agcggctccgggggcaagggaggcggaagttctgcagcggcagccgccgctgcggcagccagtggttccgga

S G S G G K G G G S S A A A A A A A A A S G S G

2665 ggcgggagttccgcagcggcagccgccgcagcagcagccagcggatcgagtggaggatccggtggatcgaag

G G S S A A A A A A A A A S G S S G G S G G S K

2737 gacaaatgcaaaagaaattccggaggatctggagccgctgctgcctccgcagctgcggccgccgccagcgga

D K C K R N S G G S G A A A A S A A A A A A S G

2809 tccaatggtaaaggttccggaagcgctgccgctgcagccgctgcggctgccgccggtggttccggaggtagc

S N G K G S G S A A A A A A A A A A G G S G G S

2881 gcttccgccgccgccgccgccgctgcagccgccggtggttccggaggctcttcttccgccgccgccgccgcc

A S A A A A A A A A A G G S G G S S S A A A A A

2953 gccgccgcggcttcgggtaagggaagctcggcgtcttccgccgccgccgcggccgccgctgctgacaaaaag

A A A A S G K G S S A S S A A A A A A A A D K K

3025 caaagcaatccaaagaaaccctgcgactactat

Q S N P K K P C D Y Y
